# Phenotypic Subtypes of Obstructive Eustachian Tube Dysfunction as Defined by Cluster Analysis

**DOI:** 10.1002/lary.70502

**Published:** 2026-03-23

**Authors:** Jenilkumar H. Patel, Duncan G. J. Green, Manal S. Malik, Steven X. Wang, Edward D. McCoul

**Affiliations:** ^1^ Department of Otolaryngology Tulane University School of Medicine New Orleans Louisiana USA; ^2^ Department of Otorhinolaryngology Ochsner Health New Orleans Louisiana USA; ^3^ Ochsner Clinical School, University of Queensland New Orleans Louisiana USA

**Keywords:** cluster analysis, endoscopy, eustachian tube, eustachian tube dysfunction, phenotype, tympanometry

## Abstract

**Objective:**

Eustachian tube dysfunction (ETD) is traditionally classified as either patulous or obstructive. Recent evidence suggests that obstructive ETD may comprise a broad array of clinical presentations. This study aimed to define subtypes of obstructive ETD according to common clinical features.

**Methods:**

This cross‐sectional study included adults diagnosed with ETD at a single academic medical center between October 2014 and June 2022. Clinical data was recorded, including patient‐reported ETDQ‐7 score, nasal endoscopy findings, tympanometry findings, medical comorbidities, timing of symptoms, symptom laterality and duration of ear fullness. Principle component analysis (PCA) was used to distinguish the most important clinical features and hierarchical cluster analysis was used to delineate symptom cluster groups.

**Results:**

Among 490 obstructive ETD patients, seven clinical characteristics were found to be the most important: duration, severity, laterality, constancy, sinusitis symptoms, history of pressure equalization tube, and comorbid reflux disease. Five phenotypic clusters were described by the clinical data, which were predictive of relative risk of comorbidity of sinusitis and GERD, as well as abnormality on endoscopy and tympanometry. Patients with bilateral symptoms had longer mean symptom duration than patients with unilateral symptoms (8.5 vs. 6.1 months; *p* < 0.001) and patients with sinusitis symptoms were more likely to have bilateral symptoms compared to those without (76.6% vs. 59.1%; OR = 0.442 [95% CI: 0.330, 0.651]; *p* < 0.001).

**Conclusion:**

Our group has identified five data‐driven symptom cluster groups that may be translated into clinical practice.

**Level of Evidence:**

3

## Graphical abstract

Obstructive ETD encompasses five clinically distinct phenotypes, ranging from mild, post‐upper respiratory infection presentations to chronic, bilateral disease driven by sinusitis and reflux. These were identified through hierarchical cluster analysis of 490 patients using seven key clinical variables. These phenotypes carry meaningful differences in objective tympanometry and nasopharyngeal endoscopy findings, suggesting individualized approaches to treatment of ETD.

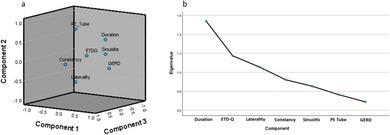

## 1 Introduction

Eustachian tube dysfunction (ETD) is a frequent cause of otologic symptoms in the general population. While highly prevalent in young children, ETD affects up to 5% of adults and accounts for over 2 million healthcare visits annually in that population [1, 2, 3]. Despite its prevalence, ETD has been typically regarded as a homogeneous condition, consisting of two broad subtypes: obstructive ETD and patulous ETD [4, 5]. More recently, the entity of nonobstructive salpingitis has been proposed [6, 7]. Nonetheless, the clinical characteristics that define individuals with ETD have not been fully studied.

Traditional assessment of ETD has relied on objective assessments with otoscopy, audiometry, impedance tympanometry, and tubomanometry [8, 9]. More recently, patient‐reported measures such as the Eustachian Tube Dysfunction Questionnaire (ETDQ‐7) [10] and systems to grade the endoscopic appearance of the eustachian tube (ET) have become available [11, 12]. Yet, current paradigms for evaluation of ETD do not take into account much of the clinical information provided during the patient interview, nor do they integrate that information with the objective measures.

Recent work in the study of chronic rhinosinusitis has utilized modern machine‐learning techniques to elucidate common groupings of characteristics based on routinely available clinical data [13, 14, 15]. In this precedent, the resulting clusters of features may be interpreted as descriptions of distinct phenotypes of disease [16, 17, 18]. A similar application of ML analysis to the clinical features of ETD has not been previously reported.

This study had several objectives. First, we sought to define naturally‐occurring groups based on clinical characteristics obtained from a large series of patients diagnosed with ETD. We then aimed to describe the common features of these phenotypic clusters. Finally, we sought to identify key clinical features that differentiate one group from another.

## 2 Materials and Methods

This was a cross‐sectional study of individuals diagnosed with ETD by a single otolaryngologist during new‐patient encounters in the outpatient clinic of an academic medical center between October 2014 and June 2022. Inclusion criteria were age 18 years or greater and a diagnosis of ETD (any type), eustachian salpingitis, and other/unspecified disorders of the ET. Exclusion criteria included nasal or nasopharyngeal tumor, central adenoid hypertrophy, and history of head and neck radiation. This study was approved by the institutional review board of Ochsner Medical Center.

Patients were asked to complete the ETDQ‐7 within 24 h prior to the clinical encounter. The ETDQ‐7 is a valid and reliable disease‐specific symptom score for adult patients, with a score range from 1 (no problem) to 7 (severe problem) [10].

Clinical data points were extracted from the electronic health record for each patient, including age, sex, the date of ETD diagnosis, duration of symptoms in months, timing of symptoms (constant vs. episodic), laterality of symptoms (unilateral vs. bilateral), and provocation of symptoms with changes in altitude (barochallenge). Duration was operationalized into three groups: < 3 months (acute), 3–12 months (chronic), > 12 months (intractable). Cardinal symptoms of sinusitis—nasal congestion, facial pressure, post‐nasal drip, and hyposmia—were recorded and summarized into a single variable representing sinusitis symptoms, in which two or more symptoms were considered positive for sinusitis while fewer than two symptoms were considered negative. Concurrent diagnoses of allergic rhinitis, acute rhinosinusitis, chronic rhinosinusitis, nasal polyps, gastroesophageal reflux (GERD), laryngopharyngeal reflux (LPR), and prior history of tympanostomy (PE) tubes. GERD and LPR were operationalized as a single variable called reflux. Patulous ETD was diagnosed by symptoms of aural fullness with autophony, a positive ipsilateral breathing test, and normal tympanometry.

Nasal endoscopy was performed on all patients. The presence of ET erythema, ET edema, posterior mucus, and tubal tonsil were recorded and summarized according to the 3ET scoring system [12]. Significant disease has been established as 3ET score > 3 out of a possible range of 0–8, with a higher value indicating more severe inflammation [11]. Impedance tympanogram was performed as part of routine evaluation and absolute tympanometric peak pressure (TPP) was recorded. A negative TPP with absolute value > 100 was consistent with a Type C curve, by convention an indicator of obstructive ETD. For supplemental analyses, a less‐negative TPP with absolute value > 100 was considered abnormal. Presence of nasal septum deviation and side of deviation (if present) were also noted.

### 2.1 Analytical Methods

Patient data were compiled and pairwise and multivariate analyses were performed to define relationships between variables. All data were analyzed in R 4.2.2 (R Foundation for Statistical Computing, Vienna, Austria). Missing data were imputed using an MCMC multiple imputation algorithm to ensure sufficient data completeness for further analyses. These include 157 (32.1%) ETDQ‐7 datapoints and 35 (7.1%) of SNOT‐22 datapoints. No other missing data were imputed due to high missingness (> 40%). To determine the most influential variables of interest for hierarchical cluster analysis, a principal component analysis (PCA) was conducted. Based on previous work the seven variables with the highest eigenvalues were selected to move forward with cluster analysis [19]. Hierarchical cluster analysis was used to describe distinct groups based on the seven most significant variables. Cluster analysis was preformed using Ward's Function and Squared Euclidean Distance. All variables were *Z*‐transformed prior to cluster analysis to account for scale differences. The effect of cluster membership was analyzed using one‐way ANOVA. Relative risk (RR) of comorbidities was calculated across all clusters for reflux and sinusitis diagnoses against the cluster with the lowest rate of diagnosis.

## 3 Results

### 3.1 General Characteristics

A total of 505 patients with ETD were included, of which 334 (66.1%) were female with a mean (SD) age of 49.1 (15.5) years (Table 1). Fifteen (3.0%) patients were diagnosed with patulous ETD and therefore were eliminated from further analyses, yielding a total of 490 patients with nonpatulous ETD for analysis.

**TABLE 1:** Descriptive characteristics of subjective clinical features for the entire study sample.

Characteristic	*n* (%)	Mean (SD)
Total	505 (100)	
Age		49.1 (15.5)
Sex		
Female	171 (33.7)	
Male	334 (66.1)	
Symptom timing		
Constant	281 (55.6)	
Intermittent	224 (44.4)	
Laterality		
Unilateral	154 (30.6)	
Bilateral	349 (69.4)	
Symptom duration, months		7.8 (7.1)
Barochallenge symptoms only	84 (16.7)	
Prior PE tubes	69 (13.7)	
Patulous ETD	15 (3.0)	
Sinusitis symptoms	297 (58.8)	
Comorbid disease		
Allergic rhinitis	145 (28.7)	
Acute sinusitis	55 (10.9)	
Chronic rhinosinusitis	78 (15.5)	
Nasal polyps	40 (7.9)	
Reflux disease	216 (44.1)	
Nasal septal deviation	239 (47.3)	
Ipsilateral	73 (30.5)	

Abbreviations: ETD, eustachian tube dysfunction; PE, pressure equalization.

Of these patients, bilateral symptoms were reported by 341 (69.6%) and constant (nonepisodic) symptoms were reported by 273 (55.7%). The mean (SD) duration of symptoms was 7.8 (4.9) months. The overall distribution of duration for the study sample was 146 (29.8%) acute, 92 (18.8%) chronic, and 251 (51.2%) intractable. Sinusitis symptoms were reported by 290 (59.2%) and 66 (13.5%) reported a prior history of PE tubes. Concurrent diagnoses included allergic rhinitis in 145 (28.7%), acute sinusitis in 55 (10.9%), chronic rhinosinusitis in 78 (15.5%), nasal polyps in 40 (7.9%), nasal septal deviation in 239 (47.3%), and reflux disease in 216 (44.1%). Objective features including ETDQ‐7 score, 3ET score and tympanometry were summarized according to standard scoring methods (Table 2). A sensitivity analysis using only participant data with complete nonimputed records showed no effect of data imputation on group characteristics. An analysis using only participant data with complete tympanometry data showed no effect of tympanometry data on group characteristics.

**TABLE 2:** Descriptive characteristics of objective clinical features for the entire study sample.

Characteristic	*n* (%)	Mean (SD)
Total	505 (100)	
ETDQ‐7 score		4.03 (1.47)
SNOT‐22 score		39.7 (19.9)
Endoscopic findings		
Total	390 (77.2)	
ET edema	174 (44.6)	
ET erythema	98 (25.1)	
Posterior mucus	117 (30.0)	
Tubal tonsil	51 (13.1)	
3ET score		2.23 (1.95)
Tympanogram		
Total	183 (36.3)	
TPP, daPa		−12.0 (58.5)
Type A	44 (56.4)	
Type B	8 (10.3)	
Type C	26 (33.3)	

Abbreviations: 3ET, Endoscopic Evaluation of the Eustachian Tube; ET: eustachian tube; ETD: eustachian tube dysfunction; ETDQ: Eustachian Tube Dysfunction Questionnaire; PE: pressure equalization; SNOT: Sinonasal Outcome Test; TPP: tympanometric peak pressure.

### 3.2 Cluster Development

**FIGURE 1:**
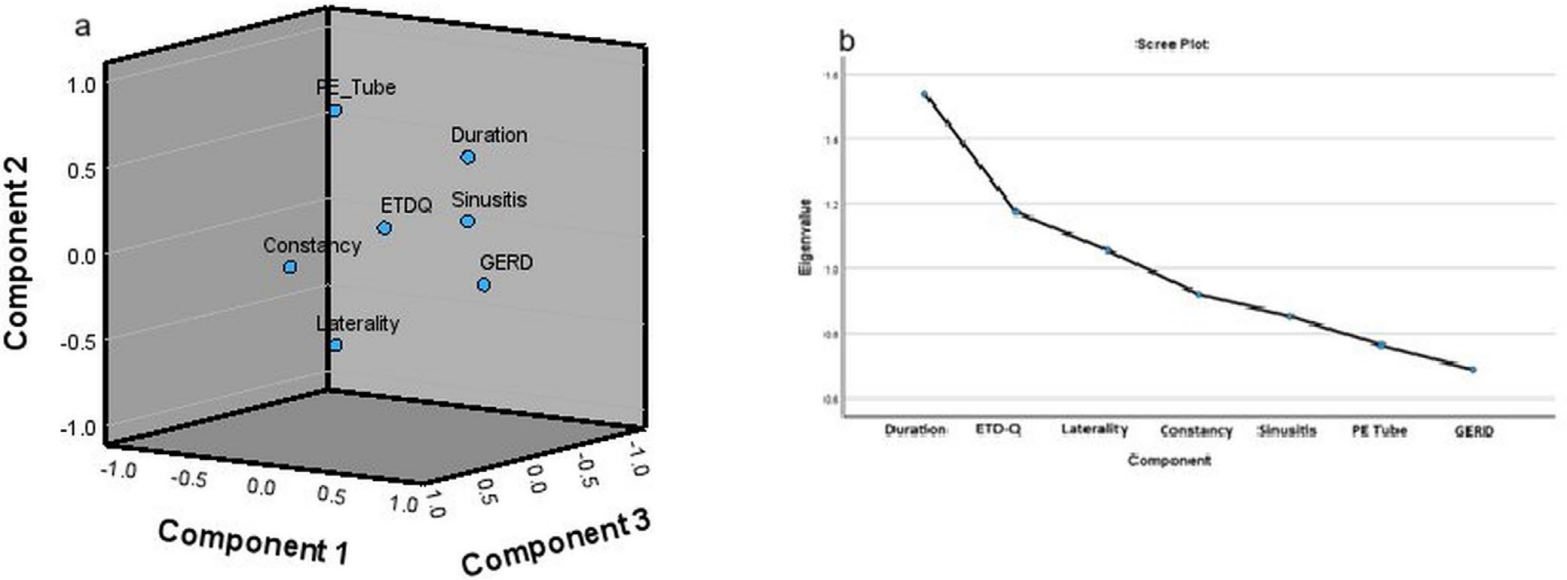
(a) Three‐dimensional scattergram visualization of principal component analysis (PCA), illustrating a spatial relationship between each variable and (b) a scree plot of eigenvalues extracted from PCA. [Color figure can be viewed in the online issue, which is available at www.laryngoscope.com]

Principal‐component analysis was conducted using all clinical variables. The seven most significant variables based on eigenvalues were severity, history of pressure equalization (PE) tubes, sinusitis symptoms, comorbid reflux disease, constancy, duration, and laterality (Figure 1a). The order of loading of each eigenvalue indicates the highest importance for symptom duration among the seven most significant variables in the PCA model (Figure 1b).

**FIGURE 2:**
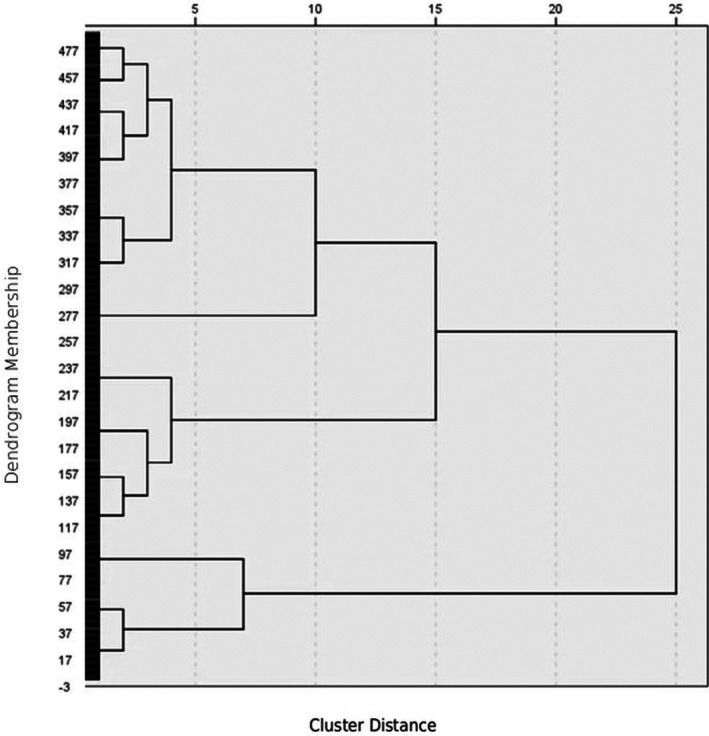
Dendrogram of divisive hierarchical cluster analysis using Ward's linkage and squared Euclidean distance. The *Y*‐axis shows overall membership of all 490 patients included at each cluster distance. The *X*‐axis shows the cluster distances between groups of patients.

The hierarchical cluster analysis generated five similar sized groups based on the seven variables of interest (Figure 2). Cluster membership was extracted and examined for group‐wise differences and commonalities. There was a significant effect of cluster membership across all seven key variables of interest (*p* < 0.05) (Table 3). Clusters were labeled based on the mean value of the most impactful variable, symptom duration, such that Cluster 1 had the briefest duration and Cluster 5 had the longest duration. The second most impactful variable, ETDQ‐7 score, was highest (6.1) in Cluster 5 and lowest (2.1) in Cluster 3.

**TABLE 3:** Cluster distribution of clinical variables with the highest contributions to cluster membership. Cluster effect determined by ANOVA. [Color table can be viewed in the online issue, which is available at www.laryngoscope.com]

	Cluster 1	Cluster 2	Cluster 3	Cluster 4	Cluster 5	Cluster effect
*N*	44	143	61	198	44	
Duration						
< 3 months (%)	86.3	74.8	0	0.5	0	*p* < 0.001
3–12 months (%)	13.6	25.2	14.8	17.2	18.2	
> 12 months (%)	0	0	85.2	82.3	81.8	
Constant symptoms (%)	81.8	60.8	44.3	51.5	47.7	*p* < 0.001
Sinusitis (%)	38.6	58	55.7	61.1	79.5	*p* < 0.05
PE tube (%)	6.8	6.9	13.1	17.7	22.7	*p* < 0.05
Bilateral (%)	5.9	35.7	21.3	25.3	20.5	*p* < 0.001
ETDQ‐7 (mean)	2.7	4.5	2.1	4.2	6.1	*p* < 0.001

*Note:* Colour coded based on severity or portion (green‐to‐red/low‐to‐high).

Abbreviation: ETDQ: Eustachian Tube Dysfunction Questionnaire.

### 3.3 Cluster Features

**FIGURE 3:**
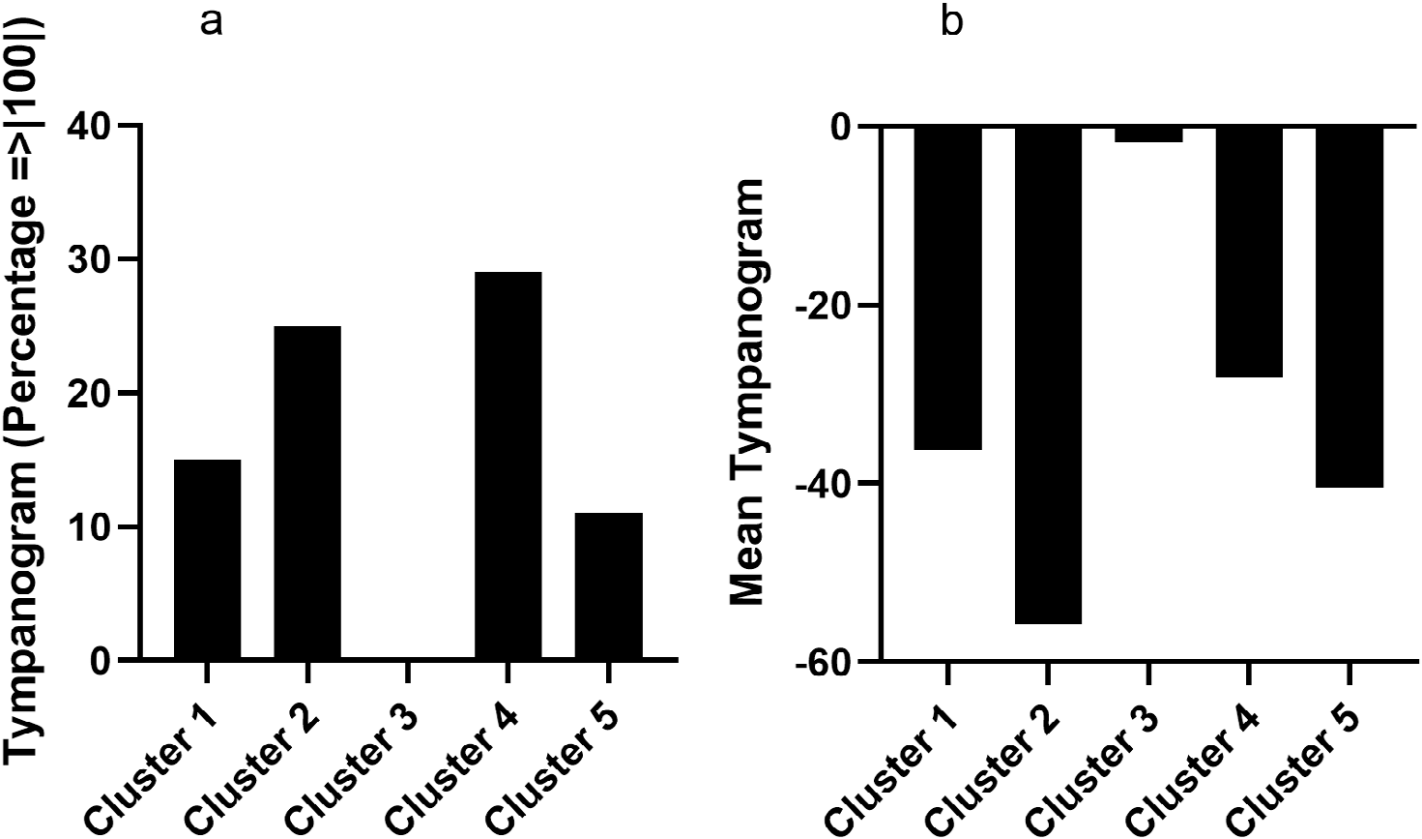
Bar chart of (a) percentage of patients per cluster with tympanogram pressure equal to, or greater than an absolute value of 100, indicating pathology and (b) mean tympanometric peak pressure per cluster.

The mean (SD) tympanometry value (TPP) was −12 (58.5), with 13.6% having a TPP absolute value > 100, indicating a Type C tympanogram. Tympanometry values differed significantly between clusters, with Cluster 4 having the largest proportion (29%) of Type C curves and Cluster 3 having the fewest (0%) (Figure 3a,b).

**FIGURE 4:**
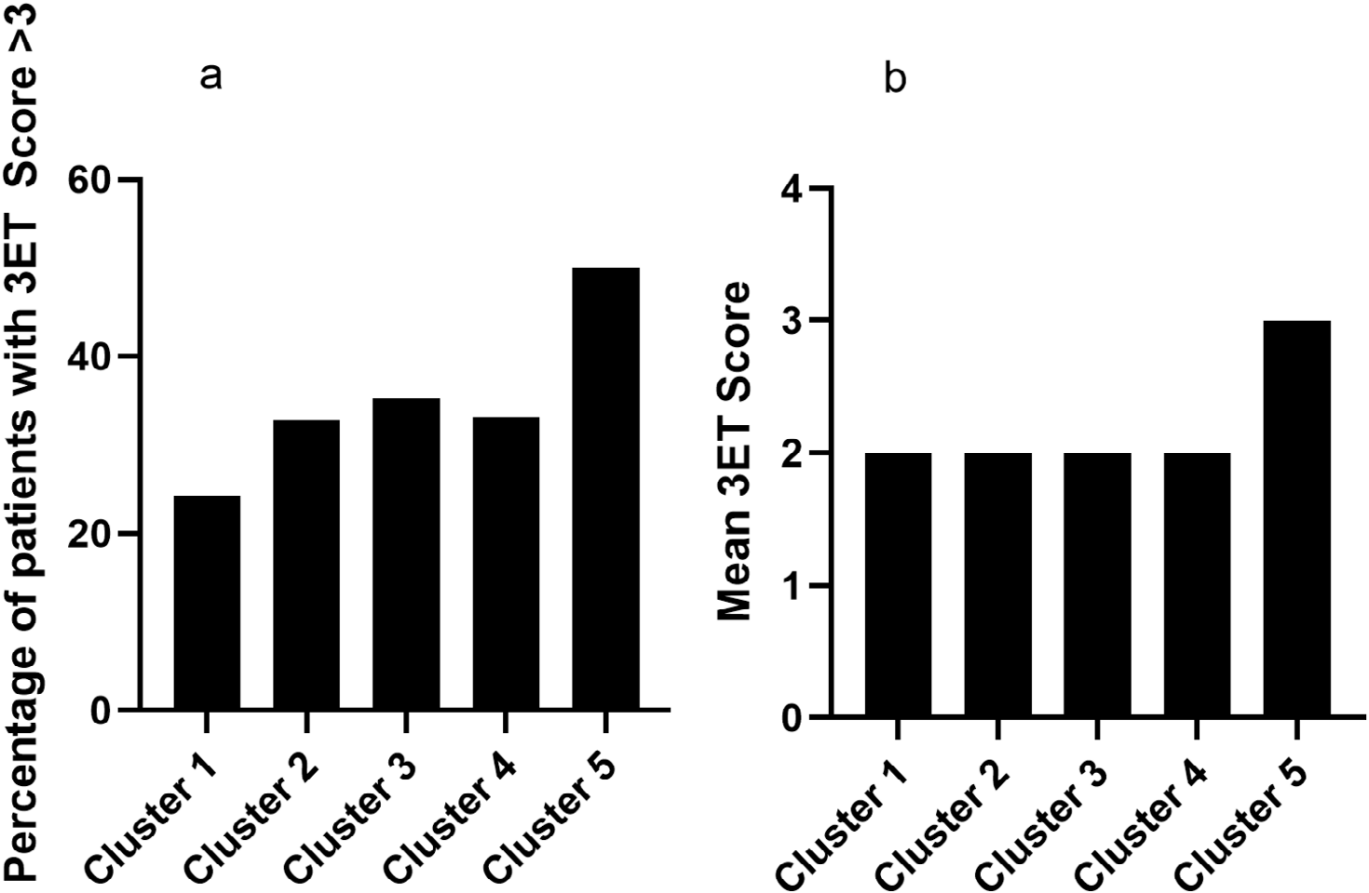
Bar chart showing a) percentage of patients per cluster with a 3ET Score > 3, indicating significant pathology and b) mean 3ET score per cluster.

The mean (SD) 3ET score was 2.3 (2.0), with 26.3% having a score > 3, indicating severe disease. The most common endoscopic abnormalities were ET edema in 174 (44.6%), posterior mucus in 117 (30.0%), ET erythema in 98 (25.1%) and tubal tonsil in 51 (13.1%). An obstructive central adenoid mass was not present in any patient. Endoscopic findings also differed between clusters, with Cluster 5 having the largest proportion (50%) of high 3ET scores and Cluster 1 having the fewest (24%) (Figure 4a,b).

Age differed significantly between clusters, with Cluster 1 being the oldest (mean 52.3 years) and Cluster 5 being the youngest (mean 44.3 years). In contrast, sex distribution did not differ significantly between clusters. The proportion of patients reporting fluctuating symptoms with barochallenge similarly did not differ significantly between clusters.

RR of comorbid reflux and sinusitis diagnoses was benchmarked against the cluster with the lowest rate of diagnosis. The RR (95% CI) was greatest for both sinusitis (2.1 [1.8–2.3]) and reflux (2.0 [1.8–2.4]) in Cluster 5.

### 3.4 Phenotype Descriptions

**FIGURE 5:**
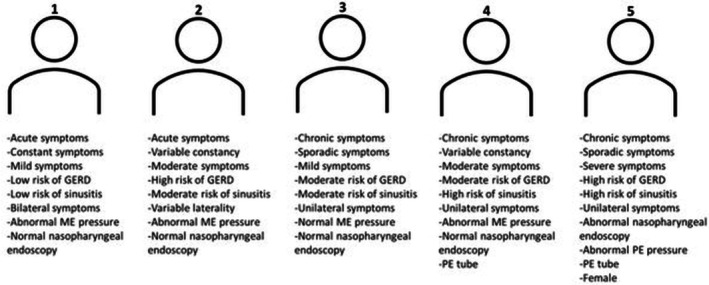
A series of patient vignettes showing the five distinct groups derived via hierarchical cluster analysis.

The differential frequency of clinical features of each cluster was sufficient to describe five distinct phenotypic groups of patients with obstructive ETD (Figure 5).

Cluster 1 was defined by acute symptoms, bilateral symptoms, constant symptoms, and objective evidence of abnormal middle ear pressure. Patients typically had less severe symptoms and were previously healthy without sinusitis symptoms or comorbid reflux disease. These were least likely to have abnormal nasopharyngeal findings on endoscopy.

Cluster 2 was defined by acute symptoms of moderate severity and was the most likely to have an abnormal tympanogram. Laterality and constancy were variable. Associations were common with reflux and sinusitis symptoms.

Cluster 3 was defined by chronic or intractable symptoms that were unilateral, episodic symptoms, and low severity. Sinusitis symptoms were common and there was typically normal tympanometry.

Cluster 4 was the largest group, defined by chronic or intractable symptoms that were unilateral, moderate severity symptoms, and concurrent sinusitis symptoms. In contrast to Cluster 3, abnormal tympanometry was common.

Cluster 5 was defined by chronic, bilateral symptoms and either constant or episodic symptoms. This group had the greatest severity of symptoms, the highest associations with reflux and sinusitis symptoms, and the highest likelihood of prior PE tubes, and the greatest female predominance of any group. This group had the greatest likelihood of abnormal nasopharyngeal findings on endoscopy and abnormal tympanometry.

### 3.5 Associations With Specific Clinical Features

Laterality of symptoms was associated with certain specific clinical features. Patients with bilateral ETD symptoms had longer mean symptom duration than patients with unilateral symptoms (8.5 vs. 6.1 months; *p* < 0.001). ETD symptoms were more often bilateral in patients with sinusitis symptoms compared to those without sinusitis symptoms (77% vs. 59%; *p* < 0.001). The frequency of bilateral ETD symptoms was comparable for patients with and without barochallenge episodes (73% vs. 69%; *p* = 0.500).

Constant (nonepisodic) ETD symptoms were equally likely in male and female patients (53% vs. 61%; OR = 0.073 [95% CI: 0.499, 1.05]; *p* = 0.095). Mean ETD symptom duration was comparable between patients with and without sinusitis symptoms (8.1 vs. 7.4 months; *p* = 0.298). Patients with sinusitis symptoms were 2.5 times more likely to have bilateral symptoms compared to those without sinusitis (76.6% vs. 59.1%; OR = 0.442 [95% CI: 0.330, 0.651]; *p* < 0.001).

In patients with nasal septal deviation, the side of deviation was not significantly associated with the occurrence of ipsilateral ETD symptoms compared to contralateral or bilateral disease (50% vs. 41%; *p* = 0.057).

Relationships were noted for ETDQ‐7 scores with several clinical features. A higher ETDQ‐7 score was associated with sinusitis symptoms compared to those without sinusitis symptoms (4.4 vs. 3.9; *p* = 0.007). A higher ETDQ‐7 score was associated with a history of PE tubes compared to no history of PE tubes (4.4 vs. 4.0; *p* = 0.049). However, ETDQ‐7 scores were not significantly different for patients with abnormal versus normal tympanograms (3.9 vs. 4.2; *p* = 0.058).

Abnormal findings on nasal endoscopy were associated with several clinical features. Comorbid reflux disease was more likely in the presence of ET edema or ET erythema compared to patients without those findings (84% vs. 72%; *p* = 0.002). Similarly, patients with reflux disease were more likely to have posterior mucus on endoscopy compared to those without reflux disease (73% vs. 60%; *p* = 0.028). Patients with CRS had less severe abnormal endoscopic nasopharyngeal findings compared to those without sinusitis symptoms (1.9 vs. 2.6; *p* = 0.012). Patients with reflux disease were twice as likely to have significant abnormal nasopharyngeal endoscopy findings compared to those without reflux (OR = 0.485; 95% CI: 0.311, 0.757).

## 4 Discussion

This study used an unsupervised machine learning approach to define phenotypic subtypes from 490 patients with non‐patulous ETD. Through PCA, seven clinical variables—symptom severity (ETDQ‐7), history of PE tubes, sinusitis symptoms, comorbid reflux, symptom constancy, symptom duration, and laterality—emerged as the most important determinants of cluster membership. Duration of symptoms was the most impactful variable, followed by ETDQ‐7 score, reflecting the role of chronicity and perceived severity in phenotypic distinction. Hierarchal clustering based on these variables resulted in five discrete phenotypic clusters, each representing a constellation of clinical characteristics. Analysis of associated clinical features revealed significant associations between longer symptom duration and bilateral involvement, higher ETDQ‐7 scores in patients with sinusitis or history of PE tubes, and differential tympanometry and nasal endoscopy findings across clusters. Clusters also varied significantly in tympanometry and 3ET endoscopy scores, supporting the presence of objective physiological correlates to subjective symptomatology. These findings suggest that, rather than a singular disease, non‐patulous ETD comprises a spectrum of phenotypes, distinguishable by a limited number of clinical characteristics.

Each cluster represents a different scenario that may occur in clinical practice. Cluster 1 is consistent with the archetypical phenotype of obstructive ETD that occurs following a viral URI, lasting only a few weeks, and which resolves spontaneously. Cluster 2 is a large cluster, which could represent an early presentation of the more chronic phenotype in Cluster 5. Cluster 3 is suggestive of symptoms that may be produced by sinonasal inflammatory disease that does not produce an objective abnormality detectable with tympanometry. This is reflected in the common occurrence of ear fullness as a symptom of both sinusitis and allergic rhinitis. Cluster 4 suggests intrinsic ET disease that is caused by neither reflux nor sinusitis. Cluster 5 is consistent with the archetypical phenotype of the chronic obstructive ETD patient with symptoms spanning many years, and suggests multifactorial etiologies from sinusitis and/or reflux. The possibility of intervention directed at the ET, such as a balloon dilation procedure, may be most appropriately considered for patients in Clusters 4 and 5, provided that extrinsic disease is controlled first. Conversely, intervention may not be advisable for Cluster 1 (due to expected self‐limited duration) and Cluster 3 (due to lack of demonstrable obstruction of the ET). Topical anti‐inflammatory treatments may be more appropriate for these patients.

This study contributes to the development of a taxonomy of ETD subtypes. Recent work has classified ETD into multiple mechanistic endotypes beyond the traditional designations of obstructive and patulous [20]. Using a combination of video endoscopy and ET function tests, Alper et al. identified six distinct ETD subtypes based on clinical symptoms, otoscopic findings, and tympanometry results. These six endotypes were designated as stricture, restriction, muscular weakness, inflammation, adhesive, and patulous/semi‐patulous. While Alper's classification emphasizes mechanistic pathways, our study enhances this framework by incorporating phenotypic clusters derived from temporal variables (symptom timing and duration), laterality, relevant comorbidities (chronic rhinosinusitis [CRS], allergic rhinitis), inflammatory findings (3ET score), and patient‐reported ETDQ‐7 scores. Additionally, rather than a broader inflammation category, our Cluster 3 highlights a unique subgroup with normal tympanometry yet significant nasopharyngeal inflammation, supporting the concept of eustachian salpingitis as a distinct clinical entity. This approach allows for a more granular differentiation of ETD phenotypes, capturing clinical heterogeneity that may guide targeted therapeutic strategies.

This study further reveals a subset of patients with ETD symptom burden despite normal tympanometry values, reflecting the emerging concept of eustachian salpingitis, or nonobstructive ETD. This term reflects a scenario of normal middle ear pressure with signs of nasopharyngeal inflammation, detectable via nasal endoscopy. This phenotype was most apparent in Cluster 3, where patients exhibited mild symptoms (ETDQ‐7 < 2.1) and normal tympanometry (mean TPP −1.8), yet elevated nasopharyngeal inflammation (3ET ≥ 3) on endoscopy. These findings support the hypothesis that non‐obstructive ETD may be driven by mucosal inflammation at the nasopharyngeal orifice of the ET rather than luminal occlusion, and suggest that tympanometry alone may be insufficient for diagnosis in this subgroup.

CRS has been previously associated with ETD, with 48%–87% of CRS patients reporting ETD symptoms [21, 22, 23]. Several prospective studies have demonstrated that endoscopic sinus surgery (ESS) for CRS leads to significant improvement in both subjective and objective measures of ET function, with normalization of tympanograms and improved Valsalva performance postoperatively [24, 25, 26]. Degree of improvement correlates with severity of preoperative sinonasal disease, and persistent ETD post‐ESS is more likely in patients with concomitant allergic rhinitis or higher baseline disease burden [27, 28]. The present study found that those with sinusitis symptoms exhibited higher ETDQ‐7 scores and increased bilateral ETD prevalence, reinforcing the established association between CRS and ETD and suggesting shared inflammatory pathways.

Nasal endoscopy is an important component for evaluating ETD, particularly for identifying inflammatory and structural abnormalities at the nasopharyngeal orifice not detected by tympanometry. The 3ET scoring system has been validated as a tool for evaluating nasopharyngeal inflammation at the ET orifice, with prior study showing a 3ET > 3 predicts symptomatic ETD in 80% of cases (specificity 97.8%, PPV 96.6%) [10, 11]. In our study, 3ET > 3 was most prevalent in Cluster 5 and least in Cluster 1, reflecting the spectrum of nasopharyngeal inflammatory burden across ETD phenotypes.

Endoscopic video analysis, including slow‐motion evaluation, is a potentially useful tool used for studying ET function. This technique distinguishes normal sequential muscle movements from pathological patterns and may be helpful in further classifying ETD [29, 30, 31]. These results underscore a role for nasal endoscopy in identifying inflammation‐driven ETD phenotypes.

This study has several limitations; our single‐center cohort study, cataloged by a single physician using consistent diagnostic criteria, enhances internal consistency but may limit generalizability due to potential clinician bias and the lack of a standardized ground truth for ETD diagnosis. Self‐reported symptom severity and comorbidities may introduce recall bias. Additionally, the cross‐sectional design precludes assessment of longitudinal outcomes or treatment responses across phenotypic clusters. Multicenter validation with standardized protocols is needed to confirm the reproducibility of our phenotypic clusters across diverse populations and healthcare settings. The cross‐sectional design precludes prognostic conclusions about treatment outcomes, such as balloon Eustachian tuboplasty for Clusters 4–5 or anti‐inflammatory therapies for Cluster 3 (non‐obstructive ETD). Prospective, longitudinal studies are recommended to evaluate therapeutic efficacy and establish differential treatment responses for these ETD phenotypes.

## 5 Conclusion

Understanding of associated clinical features may inform the establishment of more nuanced phenotypes of ETD. Laterality, symptom duration, concurrent sinusitis symptoms, reflux disease, and abnormal endoscopic nasopharyngeal findings are defining characteristics of specific phenotypes of obstructive ETD. The findings of this study may form a framework by which to establish diagnostic criteria for ETD phenotypes. Studies of prognosis and treatment may be developed utilizing the specific phenotypes of interest.

## Funding

This work was supported in part by a research grant from the Eye, Ear, Nose and Throat Foundation (EE231102).

## Disclosure

This study was presented as a poster at the Triological Society Combined Sections Meeting, West Palm Beach, Florida, January 2024.

## Conflicts of Interest

Edward McCoul (E.D.M.) is a consultant for 3D Matrix, Advanced Rx, Sanofi/Regeneron, Stryker, and Zsquare. The other authors declare no conflicts of interest.

## Data Availability

The data that support the findings of this study are available from the corresponding author upon reasonable request.
